# Interrelation of Stress, Eating Behavior, and Body Adiposity in Women with Obesity: Do Emotions Matter?

**DOI:** 10.3390/nu16234133

**Published:** 2024-11-29

**Authors:** Irene da Silva Araújo Gonçalves, Mariana De Santis Filgueiras, Tiago Ricardo Moreira, Milena Sales Thomé, Gabrielly Luisa Diniz Paiva, Geralda Patrícia de Almeida, Rosangela Minardi Mitre Cotta, Tatiana do Nascimento Campos, Dayse Mara de Oliveira Freitas, Juliana Farias de Novaes, Alex Fabrício de Oliveira, Glauce Dias da Costa

**Affiliations:** 1 Department of Nutrition and Health, Universidade Federal de Viçosa, Viçosa 36570-900, MG, Brazil; mariana.filgueiras@ufv.br (M.D.S.F.); milena.thome@ufv.br (M.S.T.); gabrielly.paiva@ufv.br (G.L.D.P.); geralda.almeida@ufv.br (G.P.d.A.); rosangelaminardi@gmail.com (R.M.M.C.); tatiana.campos@ufv.br (T.d.N.C.); dayse.freitas@ufv.br (D.M.d.O.F.); jnovaes@ufv.br (J.F.d.N.); glauce.costa@ufv.br (G.D.d.C.); 2 Department of Medicine and Nursing, Universidade Federal de Viçosa, Viçosa 36570-900, MG, Brazil; tiago.ricardo@ufv.br; 3 Centro Universitário Governador Ozanam Coelho (UNIFAGOC), Ubá 36506-022, MG, Brazil; alex.oliveira@unifagoc.edu.br

**Keywords:** stress, cortisol, obesity, eating behavior, emotional eating, women

## Abstract

Background/Objectives: Obesity is influenced by biological, hormonal, and social factors, contributing to chronic diseases and burdening the healthcare system. Chronic stress and emotional eating are linked to weight gain, affecting eating behaviors and metabolism. This study aimed to assess the association between stress, eating behavior, and adiposity in obese women. Methods: This cross-sectional study included 132 obese women from Viçosa, Minas Gerais, Brazil. The participants completed the Lipp Stress Symptoms Inventory and the Dutch Eating Behavior Questionnaire. Blood samples were collected to measure plasma cortisol, and Body Mass Index (BMI) was calculated from weight and height measurements. Body fat was assessed using dual-energy X-ray absorptiometry (DXA). Associations between stress, eating behavior, and adiposity were evaluated using linear regression models, and interactions between stress and eating behavior subscales were tested. Results: Positive associations were observed between Phases I (alert), II (resistance), and III (exhaustion) of stress with emotional and external eating. A negative association was identified between dietary restraint and body fat, especially in women with lower cortisol levels (<13.7 mg/dL). Additionally, the alert phase was associated with higher android fat in these women. Conclusions: The findings support the hypothesis that stress and eating behavior are associated with body adiposity and that stress is linked to emotional and external eating. An inverse association between restrained eating and body fat was observed in women with lower cortisol levels. These results highlight the importance of an interdisciplinary approach that incorporates emotional and stress conditions in obesity treatment.

## 1. Introduction

Obesity is a serious public health issue, particularly among women [1]. In 2023, it was estimated that 35.18% of women in Brazil were obese [2], a phenomenon that can be attributed to biological, hormonal, and social factors [3]. The increase in obesity prevalence results in a higher incidence of chronic diseases, such as type 2 diabetes mellitus, hypertension, and dyslipidemias [4], in addition to burdening healthcare systems [5].

Chronic stress can contribute to weight gain and the development of obesity [6,7,8]. The underlying mechanism involves the activation of the hypothalamic–pituitary–adrenal (HPA) axis, which results in the release of cortisol, a hormone that can promote the accumulation of abdominal fat [9]. Furthermore, stress can affect metabolism and alter how the body processes nutrients, contributing to a positive energy balance [10].

The relationship between stress and eating behavior also has direct implications for obesity [11]. Stress can lead to behavior where individuals eat to soothe emotions rather than due to physiological hunger, known as “emotional eating” [12]. This behavior is associated with increased consumption of foods high in fat and sugar, termed “comfort foods” [13].

Individuals under stress are also prone to adopting strict diets. However, dietary restriction can result in binge eating and weight gain [14]. Additionally, stress increases sensitivity to external food cues, such as the sight and smell of food, leading to excessive consumption of high-calorie foods and weight gain [15]. These stress-induced eating behaviors contribute to energy imbalance and fat accumulation, increasing the risk of obesity.

Despite advances in obesity studies, there remain gaps in approaches that consider biopsychosocial aspects in promoting nutritional care. Much of the focus on obesity revolves around food consumption in terms of quantity and quality [16,17], while minimal emphasis is placed on understanding eating behavior through dimensions such as dietary restriction, external eating, and emotional eating, as well as their relationships with stress and adiposity.

Investigating these relationships can support more effective and humanized nutritional care by considering the person as a whole and reducing reliance on approaches that contribute to weight stigma, disordered eating behaviors, and weight cycling, which in turn introduce an additional stress factor. Furthermore, exploring these interactions may help identify high-risk groups for specific nutritional interventions [18,19].

Few studies have assessed the interrelationship between stress, eating behavior, and body adiposity in women with obesity [18,20,21].

Barrington and colleagues [18] assessed the association between self-reported stress and dietary nutrient intake, examining factors such as the percentage of energy from fat, carbohydrates, and added sugar; the number of daily meals; fruit and vegetable consumption; and the intake of high-fat snacks, fast food, and sugary drinks. Analyzing data from 65,235 adults in the United States, the study found higher perceived stress correlating with increased energy intake from fat, as well as the greater consumption of high-fat snacks and fast food items.

In a review on stress and eating, Torres et al. [20] noted that stress could influence food intake in two ways, leading either to under-eating or overeating, depending on the stressor’s severity. Chronic stress, in particular, is linked to a preference for energy-dense, nutrient-rich foods, especially those high in sugar and fat. Stress-induced eating, therefore, may be an important factor in the development of obesity.

Wallis and Hetherington [21] further investigated the relationship between stress and eating in two separate studies. The first examined self-reported changes in snack intake, while the second looked at stress-induced overeating in a laboratory setting, comparing high-fat and low-fat snacks. Ninety-nine women participated, and the results indicated that the intake of high-fat snacks rose alongside higher levels of emotional eating.

Our hypothesis is that these factors interact to influence adiposity. The interaction between stress and eating behavior is complex, involving neurobiological and hormonal mechanisms that affect fat accumulation. During episodes of acute or chronic stress, the body activates the hypothalamic–pituitary–adrenal (HPA) axis, leading to the release of stress hormones like cortisol. Elevated cortisol not only promotes the intake of food rich in sugar and fat but also inhibits leptin production, the hormone that signals satiety while increasing levels of ghrelin, which stimulates appetite [22]. Moreover, stress can trigger “emotional eating”, where individuals turn to food for comfort, a behavior reinforced by the release of dopamine, a neurotransmitter associated with pleasure [23]. This craving for high-calorie foods can lead to overeating and the accumulation of visceral fat, which is closely linked to metabolic and inflammatory issues, further intensifying the stress response and creating a vicious cycle [24]. Consequently, the dynamics between stress and eating behavior contribute not only to weight gain but also to chronic inflammation, which can adversely impact both physical and mental health over the long term.

Understanding these interactions may aid in identifying high-risk target groups for differentiated nutritional interventions, providing insights that can make nutritional care more effective and humanized. The objective of this study was to evaluate the association between stress and eating behavior concerning adiposity in Brazilian women with obesity.

## 2. Materials and Methods

### 2.1. Study Design

A cross-sectional study was conducted using baseline data from an investigation that assessed the effectiveness of nutritional interventions for the health care of women with obesity. The study protocol was registered in the Brazilian Clinical Trials Registry (ReBEC) (RBR-87wb8x5). The study was approved on 10 October 2022 by the Human Research Ethics Committee of Universidade Federal de Viçosa (UFV) (protocol code 5,693,565) and conducted in accordance with the Declaration of Helsinki and the current Spanish legislation on data protection (Organic Law 3/5 December 2018, on the protection of personal data and guarantee of digital rights). Informed consent was obtained from the participants, as well as authorization to conduct the study from the Municipal Health Secretariat of Viçosa, Minas Gerais.

### 2.2. Participants

Participants underwent three different nutritional interventions in a prospective, randomized clinical trial. The recruitment of women for the study took place in the municipality of Viçosa, Minas Gerais, Brazil, from July 2022 to February 2023, using flyers, online posts via Instagram, and referrals from municipal health services.

A total of 132 adult women, aged 18 to 60 years with a Body Mass Index (BMI) greater than 30 kg/m^2^, were included in this study. Exclusion criteria were as follows: currently undergoing pharmacological treatment for obesity; having severe medical or psychiatric conditions; having insulin-dependent diabetes; being pregnant, breastfeeding, or intending to become pregnant during the study; having untreated hypothyroidism or hyperthyroidism; or having undergone bariatric surgery.

For sample power calculation, the difference between two means of independent groups was used, employing OpenEpi version 3.01. A 95% confidence interval was considered, with the number of women with grade II obesity being *n* = 32 and without obesity being *n* = 100 in the sample; there was mean and standard deviation of the Phase III stress score (exhaustion) in each of these two groups (women with grade II obesity: mean = 9.8, standard deviation = 4.6; women without obesity: mean = 6.8, standard deviation = 5.2). The calculated sample power was 87.5%.

### 2.3. Data Collection

Two meetings were held with the participants. Eligible participants attended an initial meeting for orientation and explanation about the research and subsequently attended a scheduled appointment to complete questionnaires, undergo blood collection, and receive anthropometric and body composition assessments.

Data for sample characterization were collected using a semi-structured questionnaire developed by the research team, which included questions about age, race, family income, physical activity, alcohol consumption, and smoking. The variable “hours of sleep per night” was assessed using the Pittsburgh Sleep Quality Index (PSQI) [25]. The questionnaires were administered in a private setting with only the researcher and the participant present. The research team was trained to administer the questionnaires and perform the anthropometric assessments to ensure the reliability and standardization of the collected measurements.

A pilot study was conducted with 16 women, who were not included in the final sample of the investigation, to standardize the methodology, test the instruments used, and train the nutritionists responsible for the intervention. The consultations were conducted in spaces provided by the Municipal Health Secretariat of Viçosa.

#### 2.3.1. Stress

In this present study, serum cortisol levels were used as an indicator of physiological stress. Blood samples were collected by a certified phlebotomist from a laboratory accredited by the Municipal Health Secretariat of Viçosa. The collection was performed via peripheral venous puncture, approximately two hours after the participant’s typical waking time, following a 12 h fasting period. Biochemical analysis was conducted using the Atellica^®^ CI Analyzer with reagents supplied by Siemens (Espírito Santo, Brazil).

To determine symptomatic stress, participants completed the Adult Stress Symptom Inventory (ISSL) at the time of blood collection, a validated instrument by Lipp and Guevara [26] based on a three-phase model developed by Hans Selye in 1936 [27]. The inventory provides an objective measure of stress symptoms in individuals over 15 years old and adults.

The ISSL has 37 physical and 19 psychological items, with symptoms often repeated, differing only in their intensity and severity. It has three sections corresponding to the phases of stress. The first section consists of 15 items related to the alarm phase of stress (I), characterized by physical or psychological symptoms experienced in the last 24 h. The second section refers to the resistance phase (II) and includes ten physical and five psychological symptoms experienced in the past week. The third section consists of 12 physical and 11 psychological symptoms, corresponding to the exhaustion phase (III), with symptoms experienced in the past month.

The factor analyses for the ISSL indicated a unidimensional model with strong psychometric properties (Cronbach’s Alpha Coefficient = 0.93; McDonald’s Omega Coefficient = 0.94). The factorial findings and Item Response Theory (IRT) model also demonstrated good fit (Comparative Fit Index (CFI) and Tucker–Lewis Index (TLI) ≥ 0.9; Root Mean Square Error of Approximation (RMSEA) and Standardized Root Mean Square Residual (SRMR) ≤ 0.08). Overall, the ISSL shows a robust set of psychometric evidence, supporting the validity of the interpretations proposed for its results [28].

#### 2.3.2. Eating Behavior

The participants completed the Dutch Eating Behavior Questionnaire (DEBQ) [29], translated [30] and validated [31] for Brazilian Portuguese. The DEBQ assesses the attitudes and psychosocial factors involved in food selection and decision-making. The questionnaire consists of 33 items, rated on a scale from 1 to 5, and comprises three subscales: dietary restraint (10 items), emotional eating (13 items), and external eating (10 items).

The dietary restraint subscale reflects the consistent effort an individual makes to regulate their appetite and food intake, defining an eating style associated with an awareness of proper nutritional habits. Emotional eating represents a lack of control over intake triggered by exposure to emotional stressors, leading to dietary disinhibition and reflecting a style influenced by the individual’s emotional state. External eating refers to disinhibition or a loss of control prompted by external factors, such as the food itself or the social setting in which it is consumed. The instrument does not provide specific reference values for each domain, as these are assessed on a continuous scale [30,31].

The DEBQ demonstrated psychometric properties, confirming its suitability for assessing eating behavior within the Brazilian population [30]. Expert agreement on the overall scale reached 94.50%. All DEBQ subscales exhibit satisfactory to good reliability in various subsamples [29]. Multiple international studies have reported excellent factorial validity and internal consistency for the DEBQ (α > 0.80). The three-factor structure has been confirmed in the English [32], Portuguese [31], German, French, and Swedish versions [29].

#### 2.3.3. Body Adiposity

Anthropometric assessment was conducted by a nutritionist in the anthropometry laboratory of the Health Division at UFV, following standardized guidelines [33].

Weight was measured using a digital platform scale with a capacity of 150 kg and a sensitivity of 50 g (Toledo do Brasil, model 2096PP, São Paulo, Brazil). Height was measured with the stadiometer integrated into the scale. Based on these measurements, BMI (kg/m^2^) was calculated. The values were classified as follows: grade I obesity (BMI ≥ 30 and <34.9 kg/m^2^), grade II obesity (BMI ≥ 35 and <39.9 kg/m^2^), and grade III obesity (BMI ≥ 40 kg/m^2^) [34].

Body fat (BF) was estimated using dual-energy X-ray absorptiometry (DXA; Lunar Prodigy Advance, GE Medical Systems Lunar, Milwaukee, WI, USA). For this measurement, participants wore light clothing without metal adornments and remained in a supine position on the scanner table until the scan was completed. Data on total body fat percentage and android fat percentage were estimated by the standard DXA software (version 13.31).

### 2.4. Data Analysis

The exposure variables were stress parameters (plasma cortisol; stress phases: alarm, resistance, and exhaustion) and subscales of eating behavior (dietary restraint, emotional eating, and external eating). The outcome variables were body adiposity (BMI, percentage body fat, and android fat percentage). Sociodemographic characteristics (age, race, and family income) and lifestyle variables (physical activity, insufficient sleep, alcohol consumption, and smoking) were considered covariates.

Analyses were performed using the statistical software Stata, version 14 (StataCorp LP, College Station, TX, USA). The significance level for all hypothesis tests was set at 5%. The Shapiro–Wilk test was conducted to verify data normality. Absolute and relative frequency measures were used for qualitative variables, while mean and standard deviation (SD), median, and interquartile range (IQR) were used for quantitative variables. The chi-square test for linear trend was used to evaluate relationships between qualitative variables. Analysis of variance (ANOVA) with Bonferroni post hoc or Kruskal–Wallis with Dunn post hoc was conducted to compare quantitative variables among three or more independent groups.

The interrelationship between stress, eating behavior, and body adiposity was assessed using linear regression models, reporting β coefficient estimates with their respective 95% confidence intervals (95% CI). Adjustment variables were chosen based on literature guidance. Robust variance estimates were specified in all models to account for heteroscedasticity and non-normal data distributions [35]. Interactions between stress and the eating behavior subscales were tested by incorporating an interaction term within the linear regression model. Interaction analyses were conducted exclusively on the adjusted models where significant differences were observed.

## 3. Results

The mean total body fat percentage was 49.1 ± 4.3%, and android fat was 54.0 ± 4.9%. The scores for restrained, emotional, and external eating were 2.8 ± 0.9, 3.2 ± 1.1, and 3.4 ± 0.8, respectively. Additionally, 12.9% (*n* = 17) of the women did not exhibit stress symptoms, 2.3% (*n* = 3) predominantly exhibited Phase I (alert), 42.4% (*n* = 56) exhibited Phase II (resistance), and 42.4% (*n* = 56) exhibited Phase III (exhaustion). The results indicate that the mean cortisol level was 15.6 ± 8.1 µg/dL, while stress symptom scores were 4 (IQR: 2–6) for Phase I (alert), 7 (IQR: 4–10) for Phase II (resistance), and 8 (IQR: 3–11) for Phase III (exhaustion). Women with grade III obesity exhibited higher scores for Phase II (resistance) and Phase III (exhaustion) stress symptoms compared to those with grades I and II obesity (Table 1).

When evaluating the relationship between subjective perceptions of stress and eating behavior subscales, a positive association was observed for Phases I (alert), II (resistance), and III (exhaustion) with emotional and external eating.

The statistically significant associations between stress parameters and eating behavior subdomains were as follows: Emotional eating was positively associated with cortisol levels in the unadjusted model (β = 0.02; 95% CI: 0.003, 0.04), though this association lost significance after adjustment. In both unadjusted and adjusted models, emotional eating showed a strong positive association across all stress phases (Phase I—Alert: β = 0.12; 95% CI: 0.06, 0.18; Phase II—Resistance: β = 0.12; 95% CI: 0.07, 0.16; Phase III—Exhaustion: β = 0.07; 95% CI: 0.04, 0.10). External eating was also significantly associated with stress phases in both models, particularly with Phase I—Alert (β = 0.05; 95% CI: 0.02, 0.09) and Phase III—Exhaustion (β = 0.03; 95% CI: 0.01, 0.05). No significant associations were found between restrained eating and any stress parameters in either model (Table 2).

Among the eating behavior subscales, only restrained eating showed an inverse association with body fat after adjustment (β: −1.52; 95% CI: −2.49, −0.55), with cortisol concentration as a significant interaction factor (β: 0.08; 95% CI: 0.01, 0.16) (Table 3).

**Figure 1:**
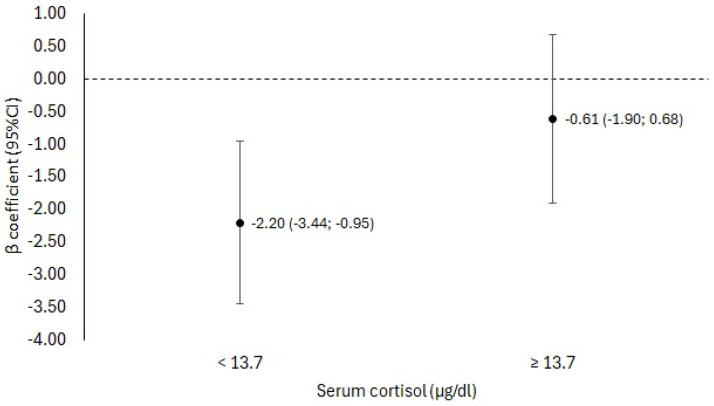
Association between restraint eating and body fat stratified by serum cortisol concentration in women from Viçosa, Minas Gerais, 2022–2023. Adjusted for age, race, family income, physical activity, insufficient sleep, alcohol consumption, smoking, alertness, resistance, and exhaustion. Serum cortisol was categorized according to the 50th percentile of the sample (13.7 µg/dL).

The inverse association between restrained eating and body fat was more pronounced in women with lower serum cortisol concentrations (β: −2.20; 95% CI: −3.44, −0.95) (Figure 1).

When evaluating the relationship of subjective perceptions of stress with body adiposity, a positive association was identified between the predominance of Phase I stress (alert) and android fat in the adjusted model (β: 3.09; 95% CI: 0.42, 5.76). No significant interaction of body adiposity with eating behavior subscales was observed (Table 4).

## 4. Discussion

Our study supports the hypothesis that stress and eating behavior are associated with body adiposity. Stress was also associated with emotional and external eating, and there was an inverse association between restrained eating and body fat in women with lower serum cortisol. Understanding these interactions can help identify high-risk target groups for differentiated nutritional interventions.

The alert state was associated with android fat. In terms of emotional aspects, the alert phase of stress is initially perceived positively, characterized by the moment when the individual gathers strength to confront or flee from stressful situations, corresponding to the coping phase [36]. From a nutritional perspective, this finding underscores the need for special care for this population, which lives under constant tension, making it difficult to initiate lifestyle changes aimed at promoting weight loss. The flight response during the alert phase leads to immediate and reactive actions that hinder conscious food choices and the adoption of sustainable dietary and lifestyle changes [37,38].

Our study corroborates the findings of Marek et al. [39], who investigated the association between psychosocial functioning (stress, anxiety, impulsivity, etc.) and weight regain, as well as other behaviors. It was observed that higher scores in stress, anxiety, and impulsivity were linked to disordered eating behaviors and weight regain after obesity treatment. Although the authors utilized a different measurement tool, the Minnesota Multiphasic Personality Inventory-3 (MMPI-3), which provides a psychometrically updated and empirically validated standard for psychological assessment, our results are consistent, suggesting that high levels of stress, anxiety, and impulsivity significantly relate to weight regain and disordered eating behaviors post-treatment.

This highlights the importance of incorporating emotional and psychosocial management in interventions to prevent relapses and promote more lasting results. It is understood that populations with these characteristics require careful attention in analyzing eating behavior, in addition to monitoring consumption and eating habits to promote a negative energy balance and weight loss [40].

Similarly, Chiu et al. [41] found that in populations living under stress due to food insecurity, there is an increased consumption of high-fat and high-sugar (hyperpalatable) foods, contributing to a greater prevalence of central obesity.

Our findings demonstrated that the three phases of stress (alert, resistance, and exhaustion) were associated with emotional and external eating in obese women. It is important to highlight that greater consumption of high-fat and high-sugar foods (hyperpalatable) was associated with higher levels of perceived stress, consequently contributing to a higher prevalence of central obesity [41].

The phases of stress—alert, resistance, and exhaustion—affect eating behavior in distinct ways, reflecting the body’s adaptive response to stress. In the alert phase, the body activates the “fight or flight” response, increasing the release of hormones such as adrenaline and cortisol. This response can suppress appetite, as the body prioritizes immediate survival and resource mobilization. During the resistance phase, the body attempts to adapt to the ongoing stressor, which can elevate cortisol levels and, in turn, increase appetite, particularly for foods high in sugar and fat, as a form of emotional compensation and energy replenishment [23]. Finally, in the exhaustion phase, where the body’s resources are depleted, many individuals may engage in “emotional eating” as a coping mechanism, seeking comfort in high-calorie foods to alleviate emotional and physical fatigue. This phase is often associated with a higher risk of overweight and obesity, as chronic stress can lead to disordered eating patterns and a preference for foods that provide temporary relief [37]. Therefore, the relationship between stress and eating behavior is complex and multifaceted, with each phase uniquely influencing individuals’ responses to food.

Furthermore, in the population with a higher proportion of android fat, it is justified to adopt stress control measures, daily organization, breathing techniques, and mindfulness, which would be more beneficial than immediately starting dietary restriction without addressing everyday stress [42,43]. Behavioral change measures associated with eating will be more effective for women who live in constant alertness to stress [44,45].

Similar studies have also found that stress is associated with emotional eating behavior [9,46], particularly in women. A cross-sectional study involving women from the United States utilizing the Three-Factor Eating Questionnaire demonstrated that perceived stress was linked to disinhibition and hunger [9]. Another study conducted with men and women from Ecuador identified a connection between perceived stress and emotional eating predominantly in women [46]. Continuous exposure to stressors and the accompanying hormonal imbalances are known to trigger an increase in appetite [47]. Ljubičić et al. [48] found associations between emotional states such as stress, depression, loneliness, boredom while eating, mindfulness during eating, and food consumption.

Building on these findings, eating in response to external stimuli was positively associated with all phases of symptomatic stress. Our results align with other authors who explain that stress intensifies the appetite for high-energy, high-calorie foods [6,7,8,49,50], leading to eating independent of physical sensations of hunger and satiety and contributing to weight gain.

We observed an inverse association between restrained eating and body fat in women with lower cortisol levels. This finding suggests that among women experiencing higher levels of stress, dietary restraint does not contribute to reducing body fat. Therefore, differentiated nutritional approaches that do not emphasize dietary restraint are necessary. Non-dietary approaches can promote healthy behavior changes, such as increased fruit and vegetable intake and enjoyable physical activity, resulting in positive impacts on anthropometric parameters, biochemical parameters, body image, depression, and stress [51,52].

Barrington et al. [18] investigated the relationship between self-reported stress and nutrient intake, including fat, carbohydrates, and sugar, in a large sample of U.S. adults. They found that higher perceived stress was associated with an increased energy intake from fats and fast food. Although we did not assess the participants’ food consumption in this study, we found a positive association between the alert phase of stress and android fat. It is well-established that a higher consumption of ultra-processed foods is linked to increased body fat, especially android fat [53]. This connection may explain the association we observed, supporting Barrington et al.’s findings.

Wallis and Hetherington [21] examined this relationship in two distinct studies. The first study analyzed changes in snack intake, while the second investigated stress-induced overeating in a laboratory setting. The results indicated that high-fat snack intake increased with higher levels of emotional eating, aligning with our findings. However, the authors also observed that food restriction decreased following stress-inducing situations in the lab. In contrast, our study found no association between food restriction and any phase of stress. This discrepancy may stem from differences in the populations studied; our study focused on women with a BMI over 30 kg/m^2^, whereas Wallis and Hetherington’s study included women with an average BMI of 21.3 kg/m^2^. For women with a higher BMI and greater obesity, food restriction and dietary adherence face numerous barriers, including individual factors such as difficulty in recognizing satiety, controlling cravings, emotional eating, and maintaining healthy eating habits. Additionally, environmental factors, such as social events with food, dining out, costs, and busy schedules, contribute to these challenges [54].

Torres et al. [20] highlighted that stress can lead to either under- or overeating, depending on the severity of the stressor. Chronic stress often promotes a preference for foods high in sugar and fat, a pattern linked to weight gain, particularly in men. Our study, conducted with women, yielded similar results, as the alert phase of stress was associated with android fat. Therefore, it appears that stress may contribute to obesity in both men and women.

In implementing nutritional strategies for stress management, it is essential to focus on organizing daily routines and using communication strategies that help women identify emotions and needs. Additionally, strategies should aim to enhance awareness of hunger, satiety, and satisfaction signals from the body [55,56,57]. Key approaches include organizing mealtime routines, creating a conducive environment for eating, focusing on the pleasure of food, and deconstructing rigid notions of “right” and “wrong” foods to help stabilize emotions and reduce stress [58]. Family support is crucial for maintaining these practices, as a supportive environment can reinforce positive behaviors [59]. Furthermore, planning techniques, such as creating shopping lists and meal plans, facilitate sustainable dietary changes [60]. Mindfulness and meditation practices, along with breathing techniques for relaxation, can also enhance individuals’ connection with their bodily signals, alleviating stress and fostering a mindful approach to hunger and satiety [61]. Finally, forming support groups can be invaluable for sustaining these changes, creating an encouraging environment conducive to stress management [62].

An interdisciplinary approach to stress management can be effectively integrated into obesity treatment by combining mindfulness techniques, psychological counseling, and nutritional education into a cohesive and practical program. For example, weekly mindfulness sessions can help participants develop awareness of their eating habits and emotions, facilitating mindful eating during meals [63]. Psychological counseling in monthly sessions can assist individuals in identifying and managing emotional triggers that contribute to uncontrolled eating [64]. Nutritional education, provided through practical workshops, can teach meal planning and healthy food preparation, fostering informed and sustainable food choices [65]. This integrated approach not only addresses the emotional and behavioral factors underlying obesity but also empowers participants to implement lasting lifestyle changes, improving stress management and reducing the risk of weight regain. This holistic integration is crucial for promoting an effective and sustainable approach to obesity treatment.

The strengths of this study include the use of validated instruments for data collection on stress and eating behavior and the use of DXA, which is considered the gold standard method for body composition analysis. The Dutch Eating Behavior Questionnaire, utilized to assess eating behavior, is among the most widely used for this purpose [66,67,68].

Regarding this study’s limitations, plasma cortisol is not regarded as the gold standard for assessing physiological stress. However, we did not use salivary cortisol in this research due to financial constraints. Blood cortisol, used as a stress indicator, has limitations in capturing daily variability in stress levels. Blood cortisol levels reflect both the circadian rhythm and the pulsatile release pattern of adrenocorticotropic hormone (ACTH), which regulates steroid production by the adrenal cortex. Cortisol peaks early in the morning and reaches its lowest levels during the night, typically between midnight and 3 a.m. However, this rhythm can be influenced by various factors, including sleep deprivation, shift work, ethnicity, gender, age, BMI, and menstrual cycle phase [69]. Certain psychobiological mechanisms that activate the hypothalamic–pituitary–adrenal axis can only be assessed indirectly through salivary cortisol measurements [70]. Despite its potential benefits, salivary cortisol analysis is costly, limiting its feasibility in clinical practice. We acknowledge that using salivary cortisol in our study could have provided a more precise assessment of stress levels. Therefore, we recommend that future research consider incorporating salivary cortisol analysis to enhance accuracy.

Additionally, this is a cross-sectional study, so causality cannot be established. Therefore, longitudinal studies are necessary for better clarity about these associations in women with obesity. Therefore, interpretations of the results should be made with caution. To validate these hypotheses, our research group conducted a longitudinal study, the results of which are currently under analysis, to track changes over time and examine the direction of these relationships. Additional studies involving repeated measurements of stress and eating behaviors at different time points could provide valuable insights into how stress influences eating over time.

## 5. Conclusions

In conclusion, stress, particularly in the alert phase, is associated with android fat in women with obesity. Additionally, all three stress phases (alert, exhaustion, and resistance) are linked to higher levels of emotional and external eating, while greater food restriction was associated with lower body adiposity only in women with lower serum cortisol levels. Our findings support the hypothesis that there is a complex interaction between stress, eating behavior, and body adiposity in women with obesity. This underscores the need for an interdisciplinary approach in their nutritional and healthcare management and the importance of actively addressing their needs. Therefore, it is crucial to have trained professionals to deliver this care, supported by public policies that strengthen integrated actions in obesity treatment for this population.

## Figures and Tables

**Table 1:** Sociodemographic characteristics, lifestyle, eating behavior, and stress parameters according to obesity in women. Viçosa, Minas Gerais, Brazil, 2023.

Variables	Obesity			*p*-Value
	Grade I (*n* = 64)	Grade II (*n* = 36)	Grade III (*n* = 32)	
Demographic and lifestyle factors				
Age (years)	38.4 (9.7)	37.5 (11.9)	35.3 (10.2)	0.39
Race (white/non-white) ^1^	46.5/49.4	32.6/24.7	20.9/25.8	0.90
Family income (BRL) ^2^	2501 (1302–3980)	2000 (1301–4953)	2604 (1100–3750)	0.89
Physical activity (no) ^1^	48.6	30.6	20.8	0.59
Insufficient sleep (<7 h) (yes) ^1^	53.1	26.6	20.3	0.24
Alcohol consumption (yes) ^1^	55.1	21.8	23.1	0.19
Smoking (yes or ex-smoker) ^1^	58.1	16.1	25.8	0.53
Eating behavior				
Dietary restraint (score)	2.9 (0.8)	2.7 (0.7)	2.9 (1.1)	0.36
Emotional eating (score)	3.1 (1.1)	3.1 (1.0)	3.4 (1.0)	0.44
External eating (score)	3.4 (0.9)	3.4 (0.8)	3.5 (0.5)	0.61
Stress parameters				
Cortisol (µg/dL)	16.0 (8.9)	14.8 (7.4)	15.7 (7.4)	0.79
Stress Phase I—Alert (score) ^2^	4 (2–5.5)	3 (2–5.5)	5 (3–6)	0.07
Stress Phase II—Resistance (score) ^2^	6 (3–9) ^a^	7 (4–10) ^a^	8.5 (7–10) ^b^	**0.01**
Stress Phase III—Exhaustion (score) ^2^	6 (3–11) ^a^	5 (1.5–11) ^a^	10.5 (7.5–13) ^b^	**0.02**

Mean (standard deviation). One-way analysis of variance (ANOVA) with Bonferroni post hoc test, *p* < 0.05. ^1^ Relative frequency. Linear trend chi-square test. ^2^ Median (interquartile range). Kruskal–Wallis test with Dunn’s post hoc test. Different letters indicate statistical significance. Bold values (*p* < 0.05).

**Table 2:** Association between stress parameters and subdomains of eating behavior in women with obesity. Viçosa, Minas Gerais, Brazil, 2023.

Stress Parameters	Eating Behavior		
	Restrained Eating β (95% CI)	Emotional Eating β (95% CI)	External Eating β (95% CI)
Cortisol (µg/dL)			
Unadjusted model	0.00 (−0.02; 0.02)	**0.02 (0.003; 0.04)**	0.01 (0.00; 0.03)
Adjusted Model	0.00 (−0.02; 0.02)	0.01 (−0.01; 0.03)	0.00 (−0.01; 0.02)
Phase I—Alert (score)			
Unadjusted model	−0.03 (−0.07; 0.02)	**0.11 (0.05; 0.16)**	**0.04 (0.003; 0.09)**
Adjusted Model	−0.02 (−0.07; 0.03)	**0.12 (0.06; 0.18)**	**0.05 (0.02; 0,09)**
Phase II—Resistance (score)			
Unadjusted model	−0.04 (−0.08; 0.00)	**0.12 (0.07; 0.16)**	**0.05 (0.01; 0.08)**
Adjusted Model	−0.03 (−0.07; 0.01)	**0.12 (0.07; 0.16)**	**0.04 (0.01; 0.07)**
Phase III—Exhaustion (score)			
Unadjusted model	−0.02 (−0.04; 0.01)	**0.07 (0.03; 0.10)**	**0.03 (0.003; 0.05)**
Adjusted Model	−0.02 (−0.05; 0.01)	**0.07 (0.04; 010)**	**0.03 (0.01; 0.05)**

95% CI: 95% confidence interval. Linear regression models with eating behavior subscales as continuous outcomes and stress markers as predictors. Robust variance estimates were specified in all models. Adjusted for age, race, family income, physical activity, insufficient sleep, alcohol consumption, and smoking. Bold values (*p* < 0.05).

**Table 3:** Association between subdomains of eating behavior and body adiposity in women with obesity. Viçosa, Minas Gerais, Brazil, 2023.

Eating Behavior	Body Adiposity		
	BMI β (95% CI)	Body Fat β (95% CI)	Android Fat β (95% CI)
Restrained eating			
Unadjusted model	−0.22 (−1.54; 1.10)	**−1.83 (−2.77; −0.88)**	**−1.03 (−2.03; −0.03)**
Adjusted model	−0.14 (−1.55; 1.27)	**−1.52 (−2.49; −0.55)**	−0.83 (−1.80; 0.15)
Restrained eating * Cortisol	-	**0.08 (0.01; 0.16)**	-
Restrained eating * Alertness	-	-	-
Restrained eating * Resistance	-	-	-
Restrained eating * Exhaustion	-	-	-
Emotional eating			
Unadjusted model	0.61 (−0.23; 1.45)	0.38 (−0.27; 1.03)	0.58 (−0.18; 1.33)
Adjusted model	0.21 (−0.86; 1.29)	−0.14 (−0.91; 0.62)	0.46 (−0.43; 1.34)
Emotional eating * Cortisol	-	-	-
Emotional eating * Alertness	-	-	-
Emotional eating * Resistance	-	-	-
Emotional eating * Exhaustion	-	-	-
External eating			
Unadjusted model	0.60 (−0.46; 1.66)	0.42 (−0.60; 1.43)	0.15 (−0.90; 1.20)
Adjusted model	0.16 (−1.07; 1.40)	−0.03 (−1.10; 1.05)	−0.12 (−1.27; 1.04)
External eating * Cortisol	-	-	-
External eating * Alertness	-	-	-
External eating * Resistance	-	-	-
External eating * Exhaustion	-	-	-

95% CI: 95% confidence interval; BMI: Body Mass Index. Linear regression models with markers of body adiposity as continuous outcomes and eating behavior subscales as predictors. Robust variance estimates were specified in all models. Adjusted for age, race, family income, physical activity, insufficient sleep, alcohol use, smoking, cortisol, alert, resistance, and exhaustion. Interactions of exposure with each stress marker in independent models adjusted for race the same variables. Bold values (*p* < 0.05).

**Table 4:** Association of cortisol and predominant stress phases with body adiposity in women with obesity. Viçosa, Minas Gerais, Brazil, 2023.

Stress Parameters	Body Adiposity		
	BMI β (95% CI)	Body Fat β (95% IC)	Android Fat β (95% CI)
Cortisol (µg/dL)			
Unadjusted model	0.02 (−0.10; 0.14)	0.04 (−0.03; 0.11)	0.05 (−0.03; 0.13)
Adjusted model	0.01 (−0.12; 0.14)	0.03 (−0.04; 0.10)	0.05 (−0.04; 0.15)
Cortisol * Restrained eating	-	-	-
Cortisol * Emotional eating	-	-	-
Cortisol * External eating	-	-	-
Predominant stress phases ^1^			
I—Alertness			
Unadjusted model	3.03 (−0.03; 6.10)	−0.64 (−5.87; 4.58)	**3.37 (0.31; 6.43)**
Adjusted model	2.93 (−1.81; 7.67)	−0.01 (−3.55; 3.53)	**3.09 (0.42; 5.76)**
Alertness * Restrained eating	-	-	0.72 (−1.32; 2.76)
Alertness * Emotional eating	-	-	1.19 (−1.90; 4.27)
Alertness * External eating	-	-	5.60 (−2.91; 14.11)
II—Resistance			
Unadjusted model	0.89 (−1.24; 3.02)	0.07 (−2.04; 2.18)	0.76 (−1.59; 3.11)
Adjusted model	0.69 (−1.66; 3.03)	−0.27 (−2.42; 1.89)	0.48 (−1.77; 2.73)
Resistance * Restrained eating	-	-	-
Resistance * Emotional eating	-	-	-
Resistance * External eating	-	-	-
III—Exhaustion			
Unadjusted model	1.70 (−0.75; 4.16)	−0.48 (−2.63; 1.67)	0.61 (−1.70; 2.93)
Adjusted model	1.89 (−0.71; 4.50)	−1.05 (−3.21; 1.10)	0.59 (−1.62; 2.81)
Exhaustion * Restrained eating	-	-	-
Exhaustion * Emotional eating	-	-	-
Exhaustion * External eating	-	-	-

95% CI: 95% confidence interval; BMI: Body Mass Index. Linear regression models with markers of body adiposity as continuous outcomes and stress markers as predictors. Robust variance estimates were specified in all models. Adjusted for age, race, family income, physical activity, insufficient sleep, alcohol use, smoking, restrained eating, emotional eating, and external eating. Interactions of exposure with each eating behavior subscale in independent models adjusted for race the same variables. ^1^ The category “no stress” was considered the reference. Bold values: *p* < 0.05.

## Data Availability

The data presented in this study are available on request from the corresponding author. The data contain sensitive information that could compromise participant privacy, infringing upon ethical considerations and Brazilian data protection laws.
